# Emojis as socio-emotional cues in EFL learning: a narrative conceptual review of affect, relatedness, and well-being

**DOI:** 10.3389/fpsyg.2026.1873670

**Published:** 2026-10-16

**Authors:** Shamim Akhter, Abdulrazaq Alzahrani, Rabindra Dev Prasad, Musarat Shaheen

**Affiliations:** 1 INTI International University, Nilai, Negeri Sembilan, Malaysia; 2 Umm Al-Qura University, Makkah, Saudi Arabia; 3 Bahauddin Zakariya University, Multan, Pakistan

**Keywords:** digital semiotics, EFL learning, emojis, narrative review, positive psychology, psychological well-being, self-determination theory, socio-emotional cues

## Abstract

Emojis can modify the emotional and relational interpretation of digital messages, but their contribution to learner well-being in English as a Foreign Language (EFL) education remains uncertain. This narrative conceptual review brings together purposively selected research on emojis, educational communication, and language learner emotions. It distinguishes direct emoji evidence in EFL from emoji research in other settings, earlier emoticon studies, and contextual evidence on digital learning without an isolated emoji component. Self-determination theory provides the principal account of how perceived support might influence relatedness and motivation; digital semiotics explains cue interpretation, while broaden-and-build theory and a multidimensional view of well-being delimit possible affective processes and distal outcomes. The proposed model connects an emoji and its accompanying text to appraisals of communicative intent, warmth, appropriateness, and competence. These appraisals may influence affect and relatedness, with subsequent implications for willingness to communicate and engagement. Culture, familiarity, message purpose, sender identity, and relationship history condition these links. Selected studies support specific effects on interpretation, impressions, self-efficacy, or immediate task performance; they do not establish that emojis produce enduring well-being or resilience in EFL learners. The review develops testable propositions, considers adverse and null effects, and offers context-sensitive pedagogical implications. Its contribution is a bounded account of how small instructional cues might matter and what evidence is required to establish their consequences.

## 1 Introduction

The emergence of digital communication technologies has altered the landscape of language use, blurring boundaries between spoken and written modalities and generating new hybrid communicative norms. Among the features of contemporary digital discourse is the emoji, a pictographic symbol transmitted through digital platforms to convey emotional, social, and contextual meaning. Bai et al. (2019) reviewed emoji research across disciplines and documented its semantic, emotional, and pragmatic functions. Its use in educational communication raises questions about how learners interpret messages and whether those interpretations affect their participation.

For English as a Foreign Language (EFL) learners, who frequently navigate the affective challenges of communication in an additional language, emojis may hold particular significance. Language learning is an inherently emotionally laden enterprise (Dewaele and MacIntyre, 2014); learners experience foreign language anxiety, frustration, embarrassment, and self-doubt alongside achievement, curiosity, and social belonging. MacIntyre and Gregersen (2012) developed a theoretical argument for the positive-broadening role of emotion and imagination in language learning. Their article provides a rationale for investigating supportive communication, rather than experimental proof that any particular digital cue builds durable psychological resources.

Text-based instruction lacks some of the vocal and bodily cues available in face-to-face interaction, but it is not inherently emotionally impoverished. Walther's (1992) account of computer-mediated communication explains how people can develop relational information through the cues available to them. Emojis are one such resource, alongside wording, punctuation, response timing, and interaction history. Their potential contribution must therefore be evaluated against an appropriate textual comparison, rather than against an assumption that messages without emojis cannot express care.

Existing studies address different parts of this problem. Chen and Hsu (2022) investigated emoji feedback and EFL self-efficacy; Sia et al. (2024) examined perceived instructor credibility and motivation in higher education; and Li and Zhong (2025) tested immediate semantic matching among English-speaking learners of Chinese. These studies differ in population, communicative function, and outcome. The theoretical gap is the absence of a sufficiently specified account linking the interpretation of a small instructional cue to distinct affective and relational processes, and then to possible educational outcomes, without treating these levels as interchangeable.

Contemporary work also situates affective support within technology-mediated EFL learning. Zhang and Yang (2026) reported improved speaking outcomes and reduced anxiety following a 16-week programme combining appreciative education with oral training assisted by artificial intelligence (AI). The intervention phase involved 13 participants in a single-group pretest–posttest pilot; its reflective data also identified psychological safety as a theme. This provides relevant context for supportive digital pedagogy, but it neither isolates emojis nor establishes the causal contribution of individual programme components. The unresolved question for the present review concerns the micro-level cues embedded in everyday instructional messages.

We pursue three primary objectives: (1) to characterize the semiotic and paralinguistic properties of emojis as communicative resources; (2) to distinguish the evidence for specific emoji-related outcomes from theoretical extensions to learner well-being; and (3) to propose a coherent framework, testable research agenda, and context-sensitive pedagogical implications. Well-being is treated as a possible distal consequence requiring its own evidence, rather than as an established effect of emoji use.

## 2 Review approach and evidence boundaries

### 2.1 Scope and literature identification

This article is a narrative conceptual review. Its purpose is to develop and critically delimit an explanatory model, rather than to estimate an overall intervention effect or map all available studies. Literature was selected purposively for its relevance to emoji interpretation, instructional communication, language learner emotion, and the theoretical links among these domains. The purposive selection does not establish exhaustive coverage of the literature.

The targeted literature update was conducted on 22 September 2026 using targeted web searches, exact-title and DOI searches for cited studies, and reference tracing from relevant publications. Search terms combined emoji or emoticon with EFL, instructor, feedback, educational communication, learner emotions, and well-being. Title-based searches were also used to retrieve work on instructor impressions and individual differences in emoji comprehension. Publisher pages, available article texts, scholarly repository copies, and bibliographic records were consulted to check study design, population, measured outcomes, and citation details. Recent empirical studies published from 2022 to 2026 were prioritized for the update, while earlier publications were retained where they supplied foundational theory or relevant counterevidence. The update was intended to refine the conceptual synthesis rather than establish exhaustive coverage.

Eligible contributions were peer-reviewed empirical studies directly examining emojis or emoticons, research on affective and relational processes in language or educational communication, and scholarly theoretical works needed to define the proposed mechanisms. Studies outside EFL were retained when they informed cue interpretation or a contextual boundary, with their transfer limits stated. Sources about digital interaction without an identifiable emoji manipulation or measure were used only for background. Sources were used only for claims supported by their reported design and findings. Selection was based on conceptual relevance and evidential fit, not on whether a study reported a favourable effect.

### 2.2 Synthesis and appraisal

The synthesis distinguishes four evidence categories: direct emoji research in EFL; emoji research in other educational, language-learning, or communication settings; related emoticon research; and contextual research without an isolated emoji component. Foundational theories provide explanatory resources but are not counted as empirical confirmation of emoji effects. Table 1 presents selected studies that anchor these distinctions; it is an illustrative evidence map, not a complete inventory.

**Table 1:** Selected empirical studies and the boundaries of their relevance to the proposed model.

Source and evidence category	Design and context	Relevant finding	Inference boundary
Chen and Hsu (2022) direct EFL emoji evidence	Three studies: survey (*n* = 310), feedback study (*n* = 120), and follow-up (*n* = 35).	Positive emoji feedback supported self-efficacy; learners also reported preferences for emoji feedback.	Self-efficacy and preferences do not establish relatedness mediation or sustained well-being.
Vareberg et al. (2023) other educational emoji evidence	Factorial welcome-email experiment; 368 students.	Higher caring, immediacy, and liking impressions accompanied lower competence and trustworthiness impressions.	Immediate instructor impressions; not an EFL anxiety or well-being intervention.
Sia et al. (2024) other educational emoji evidence	Observational survey; 348 Malaysian higher-education students.	Associations linked reported emoji use, instructor credibility, motivation, and perceived performance.	Self-report and observational mediation cannot establish a causal instructional effect.
Blobstein et al. (2025) other educational emoji evidence	Collaborative reading annotations from over 1,800 biology students.	Emoji–hashtag pairs expressed questions and reactions and helped instructors inspect student responses.	Student expression and instructor interpretation; no isolated effect on EFL emotions or well-being.
Li and Zhong (2025) other L2 emoji evidence	Within-subject semantic matching; 30 English-speaking learners of Chinese.	Congruent emoji cues facilitated immediate matching relative to the verbal comparison.	Not an EFL sample; no delayed acquisition or socio-emotional outcome.
Chen et al. (2024) other-context emoji evidence	Facial-emoji classification across platforms; 523 UK and Chinese participants.	Classification varied with culture, age, and gender.	Decontextualized emotion labels do not establish classroom-message interpretations.
Derks et al. (2008) related emoticon evidence	Message-interpretation experiment; 105 secondary-school students.	Emoticons intensified messages; conflicting valence could create ambiguity or sarcasm.	Emoticons, not contemporary emoji repertoires; no EFL well-being outcome.
Glikson et al. (2018) related pictorial-smiley evidence	Three experiments on virtual work-related first impressions.	Smileys could reduce competence impressions without a reliable warmth benefit.	Workplace context; relevant to appropriateness, not direct classroom effects.
Han et al. (2024) contextual non-isolated evidence	Mixed-methods study; 80 intermediate EFL learners in four intact classes.	Digital communication activities were associated with gains in WTC and engagement.	The intervention did not isolate emojis.
Zhang and Yang (2026) contextual non-emoji evidence	16-week single-group intervention pilot; 13 EFL learners.	Speaking outcomes and anxiety improved; reflections described psychological safety.	Appreciative education and AI-assisted practice were combined; no emoji effect or component causality established.

Sample sizes are those reported in the cited publications. The rows are illustrative rather than exhaustive. L2, second language; WTC, willingness to communicate.

For each focal study, the synthesis considers the cue and sender, population and setting, design, measured outcome, and scope of inference. Findings are compared in terms of what they establish about interpretation, immediate impressions, affect, motivation, or behaviour. The appraisal is qualitative and attends to self-report, observational versus experimental design, bundled interventions, and the presence or absence of follow-up. The review did not use formal risk-of-bias scoring, independent dual screening, or quantitative pooling. Accordingly, the resulting propositions are hypotheses to be tested, not conclusions from a meta-analysis.

## 3 Emojis as paralinguistic and semiotic resources

### 3.1 What makes an emoji a socio-emotional cue

From a semiotic perspective, emojis may operate iconically by resembling a face or object, indexically by suggesting a stance, and symbolically through learned conventions. These functions do not make their interpretation universal or transparent. Danesi (2017) places emojis within the development of visual communication, while Herring and Dainas (2017) examine graphicons in interaction. Together, these perspectives direct attention to the relationship between an image, its textual surroundings, and its use by particular participants.

An emoji functions as a socio-emotional cue when its placement helps a recipient infer how a message should be understood and what relational stance the sender is adopting. A picture used to name an object in a vocabulary task serves a different function from a smiling face accompanying corrective feedback. The latter may qualify the perceived force or interpersonal tone of the correction without changing its propositional content. Thus, the relevant unit is the emoji–text–sender configuration in context, not the isolated symbol.

Consider the illustrative instruction, “Please revise the final sentence.” Adding a smiling-face emoji could suggest encouragement, but it could also appear patronizing if the learner has already expressed frustration. A wink could invite a playful reading or introduce uncertainty about whether the request is serious. These are constructed examples of possible interpretations, not observations from participants. They illustrate why positive visual valence cannot be equated with positive psychological impact.

Facial emojis are often used to suggest affective stance; gestures can acknowledge a contribution or signal evaluation; and objects or symbols may supply referential meaning, mark completion, or celebrate an achievement. These categories overlap in use. A check mark may be understood as task confirmation rather than interpersonal warmth, and a celebration symbol may suggest encouragement without conveying specific information about performance. Comparisons should therefore specify the chosen cue, its position, and its intended function instead of treating all emojis as an equivalent intervention.

### 3.2 Interpretation precedes psychological response

The concept of digital paralanguage provides a productive lens for understanding how visual cues accompany and modify verbal content. Emojis can help a recipient attribute sincerity, humour, approval, or criticism to a message; the recipient then evaluates what that stance implies for the relationship and the task. In the proposed mechanism, noticing the cue and interpreting its appropriateness precede an appraisal of warmth or threat. A smile interpreted as sarcasm can therefore reverse the expected direction of an affective response.

Earlier emoticon experiments are relevant but should not be relabelled as evidence about contemporary emoji repertoires. Derks et al. (2008) showed that emoticons could intensify message interpretation and that disagreement between verbal and visual valence could create ambiguity or sarcasm. Riordan (2017) provides emoji-specific evidence concerning communicated affect in text messages. These findings support attention to message interpretation; they do not by themselves establish reduced EFL anxiety or improved well-being.

Emojis may provide additional resources for learners whose second-language (L2) pragmatic knowledge is still developing. However, learner status should not be taken to imply that non-native writing is inherently rude, or that visual supplementation automatically develops pragmatic competence. Any proposed scaffolding benefit requires evidence that learners interpret the cue as intended and can use that understanding in later communicative tasks.

### 3.3 Sender roles and automated feedback

Instructor-produced emojis occur within an evaluative relationship. A cue may soften the perceived interpersonal tone of feedback while leaving its accuracy and instructional usefulness unchanged. Learner-produced emojis may instead express stance, request affiliation, manage uncertainty, or negotiate peer-group norms. The communicative meaning of a learner’s reply cannot be inferred solely from the instructor’s intended tone, and evidence from instructor messages should not be generalized automatically to peer interaction.

Recent educational research illustrates this learner-side function. Blobstein et al. (2025) analysed student use of instructor-selected emoji–hashtag pairs in collaborative annotations of biology readings. The cues helped make students’ questions and reactions visible to instructors. This is evidence about the expression and interpretation of learning experiences, not an intervention showing that emoji use improves emotions or well-being.

A further distinction concerns system-delivered feedback, including preprogrammed responses and AI-generated messages. The emojis embedded in computer-assisted language learning materials studied by Chen and Hsu (2022) provide evidence about feedback design, not evidence that generative AI conveys genuine care. A learner may respond differently to the same cue when it is attributed to a teacher, a peer, or an automated system. Sender attribution, perceived responsiveness, and awareness of automation should therefore be specified in both theory and research design. Survey evidence on AI tools in higher education points in a similar direction. Among 805 Indonesian university students, Habibi et al. (2026) found that instructor quality and knowledge acquisition, sharing, and application predicted satisfaction with AI tools, whereas the content quality and technical system quality of the tools did not. That study did not examine emojis or feedback wording, but it indicates that learners’ responses to technology-mediated support depend on the human instructional context in which the technology is embedded.

## 4 Positive psychology and learner emotions

### 4.1 Distinguishing the constructs

Positive psychology, established as a formal discipline by Seligman and Csikszentmihalyi (2000), redirected psychological inquiry toward the study of positive functioning as well as difficulties. Within applied linguistics, this perspective broadened attention to enjoyment, relationships, and the conditions supporting participation (MacIntyre and Mercer, 2014; MacIntyre et al., 2019). It does not require an assumption that positive and negative emotions are mutually exclusive, or that all supportive-looking communication is beneficial.

Positive affect refers here to a relatively immediate emotional experience, such as enjoyment or reassurance. Perceived warmth is an appraisal of another person’s communicative stance; relatedness concerns feeling connected and cared for. Motivation concerns the direction and regulation of activity, whereas academic engagement concerns involvement in learning, including behavioural participation, cognitive investment, and emotional involvement. Willingness to communicate (WTC) is readiness to initiate L2 communication in a particular context; it is distinguishable from both actual participation and linguistic achievement. Self-efficacy is a belief about one’s capability to perform a task, not a direct measure of competence or well-being.

Learner well-being concerns broader positive functioning and experience across time and contexts. Psychological resources, such as coping capacities or supportive relationships, must also be specified and measured rather than inferred from an immediate favourable response. The distinction matters because a message may make a teacher seem friendlier without changing the learner’s anxiety, sustained engagement, or overall well-being. Dewaele and MacIntyre (2014) and Dewaele et al. (2023) support differentiating language-learning emotions; they do not test emoji effects.

### 4.2 Self-determination theory as the principal foundation

Self-determination theory (SDT; Deci and Ryan, 1985; Ryan and Deci, 2000) identifies autonomy, competence, and relatedness as basic psychological needs relevant to motivation and well-being. It supplies the principal motivational explanation in the proposed model: if an instructional cue is interpreted as responsive and respectful, it may contribute to perceived relatedness, which may in turn support more autonomous participation. This is a conditional application of SDT, not a claim that SDT has already validated an emoji intervention.

Relatedness is the most immediate need implicated by the socio-emotional function examined here. Competence support depends on informative, usable feedback and opportunities for successful action; a congratulatory symbol alone does not demonstrate competence support. Autonomy requires meaningful choice and acknowledgement of learners’ perspectives. Merely allowing emoji selection is insufficient evidence that this need has been satisfied. Conversely, expectations that students display cheerful reactions may be experienced as controlling and could undermine autonomy.

### 4.3 Supporting perspectives and their limits

Digital semiotics explains how the cue acquires contextual meaning before it can enter the psychological pathway. Sociocultural theory situates such meaning-making within mediated interaction and shared practices (Vygotsky, 1978). These perspectives explain the communicative setting and interpretive process; they are not additional parallel theories of well-being in the model. Shared practices can develop within learning communities (Wenger, 1998), but shared emoji use is not itself evidence of equitable inclusion or effective learning.

Fredrickson’s (2001) broaden-and-build theory supplies a secondary affective hypothesis: when an interaction actually elicits positive emotion, that experience may facilitate exploratory action and, through repeated experiences, contribute to resource development. The theory does not establish the preceding link from emoji presence to positive emotion. Nor does an immediate affective response demonstrate that resources have accumulated. In this review, broaden-and-build therefore motivates a temporal extension to investigate, rather than a conclusion about resilience or altered emotional set-points.

Seligman’s (2011) PERMA model identifies positive emotion, engagement, relationships, meaning, and achievement as dimensions relevant to well-being. Here, PERMA is an outcome-organizing framework, not a second causal mechanism. The reviewed emoji studies are most directly relevant to selected emotional and relational responses. Meaning and achievement require independent evidence, and improvements in one dimension should not be treated as equivalent to overall flourishing. Oxford’s (2016) broader account of language learner well-being likewise highlights that such functioning cannot be reduced to a single communicative feature.

### 4.4 What the empirical literature supports

The most directly relevant EFL evidence concerns self-efficacy rather than well-being. Chen and Hsu (2022) combined a survey, an emoji-feedback study, and a smaller follow-up investigation. Their findings support the possibility that positive emoji feedback in computer-assisted language learning can influence EFL learners’ self-efficacy. They do not demonstrate the complete sequence from perceived teacher warmth to relatedness, reduced anxiety, and enduring well-being. The distinction between confidence in learning and measured learning outcomes remains necessary.

Evidence from higher education also shows why outcomes should be separated. Sia et al. (2024) reported relationships among perceived emoji use, instructor credibility, motivation, and learning performance in survey data from 348 Malaysian higher-education students. Because these were self-reported, observational relationships, the results do not establish causal mediation or an emoji-specific effect on EFL anxiety. In contrast, the welcome-email experiment reported by Vareberg et al. (2023), with 368 students, found more favourable caring, immediacy, and liking impressions alongside lower competence and trustworthiness impressions. Relational warmth and perceived instructional authority can therefore move in different directions.

Cognitive findings address another outcome. Li and Zhong (2025) found an advantage for semantically congruent emoji cues in an immediate matching task with 30 English-speaking learners of Chinese. The population was not an EFL sample, and the task did not test durable vocabulary acquisition or socio-emotional mediation. This study supports a bounded hypothesis about visual-semantic processing, which should remain distinguishable from the affective and relational pathways.

Finally, broader digital-learning findings provide context. Han et al. (2024) reported changes in WTC and engagement following digital communication activities, but their intervention did not isolate an emoji effect. Alrabai (2024) examined relationships among emotions, motivation, and WTC among Saudi EFL learners without testing emojis. Akhter and Shaheen (2026) reported, in cross-sectional survey data from 482 university students in Pakistan, that AI adoption was associated with academic motivation and well-being, and that the association with well-being was stronger among students reporting greater peer support. Their design neither tested emojis nor established causal direction, but the moderating role of peer support is consistent with locating relatedness in social relationships rather than in the technology itself. These studies support the relevance of the proposed outcomes and relationships, while leaving the incremental contribution of an emoji unresolved.

## 5 Cultural and contextual boundary conditions

### 5.1 Culture in the interpretive mechanism

A critical caveat to any account of emojis as socio-emotional resources is variability in their interpretation across cultural, generational, and individual differences. Emoji meaning is not fixed; it is co-constructed within social and cultural contexts of use, and the same symbol may carry different connotations for different users. Platform rendering, familiarity, local conventions, and the history of an exchange can influence interpretation (Bai et al., 2019).

Chen et al. (2024) examined facial-emoji classification among 523 participants in the United Kingdom and China and found differences associated with culture, age, and gender. Their classification task does not establish how every learner in either country interprets a teacher’s message. It does, however, show why researchers should measure interpretive variation rather than assume that a nominally positive cue has a shared emotional meaning. National categories are an incomplete proxy for the communicative practices of particular learners.

Culture is therefore positioned as a boundary condition at the first step of the model: it may shape how an emoji–text combination is interpreted, which can alter subsequent appraisals of warmth, sincerity, and appropriateness. Norms about teacher authority and acceptable informality may also condition how an appraisal translates into willingness to respond. These are hypotheses about specific links, not fixed predictions about cultural groups. Studies should examine within-group differences and measure relevant norms and familiarity directly.

### 5.2 Adverse and unintended consequences

An emoji can introduce ambiguity when its apparent emotion conflicts with the text, or when a recipient interprets it as irony, dismissal, or criticism. Overuse may make feedback seem formulaic, while a playful cue accompanying a serious concern may appear insensitive. A smiling face does not necessarily soften a correction, and an apparently encouraging gesture may be read as pressure to comply. These possibilities should be evaluated alongside neutral responses and beneficial ones.

Evidence outside education reinforces this boundary. Glikson et al. (2018) found that pictorial smileys in work-related first impressions could reduce perceived competence and did not reliably increase warmth; appropriateness was important to interpretation. This finding cannot determine classroom effects, but it directly challenges a general assumption that adding a smiley necessarily humanizes communication. The mixed impression results of Vareberg et al. (2023) establish a corresponding concern within educational communication.

Relationship history, message stakes, proficiency, baseline anxiety, and accessibility may further alter responses. Low-stakes peer exchanges and formal grading messages have different expectations. Students may also feel pressure to reciprocate an instructor’s expressive style. Where rendering or assistive technology makes a cue difficult to interpret, the intended relational function may be lost. A supportive environment must allow students to communicate comfortably without requiring emoji use or interpreting its absence as disengagement.

## 6 A conditional model of emoji-mediated instructional communication

The proposed model begins with an emoji embedded in a particular message and attributed to a particular sender (Figure 1). The recipient interprets the cue in relation to the text, message purpose, relationship, and local conventions. This interpretation informs appraisals of communicative intent, warmth, appropriateness, and competence. A positive appraisal is possible, but so are ambivalence, misunderstanding, and no meaningful change. Culture and familiarity enter at this interpretive stage rather than appearing only as limitations after the proposed effects.

**Figure 1:**
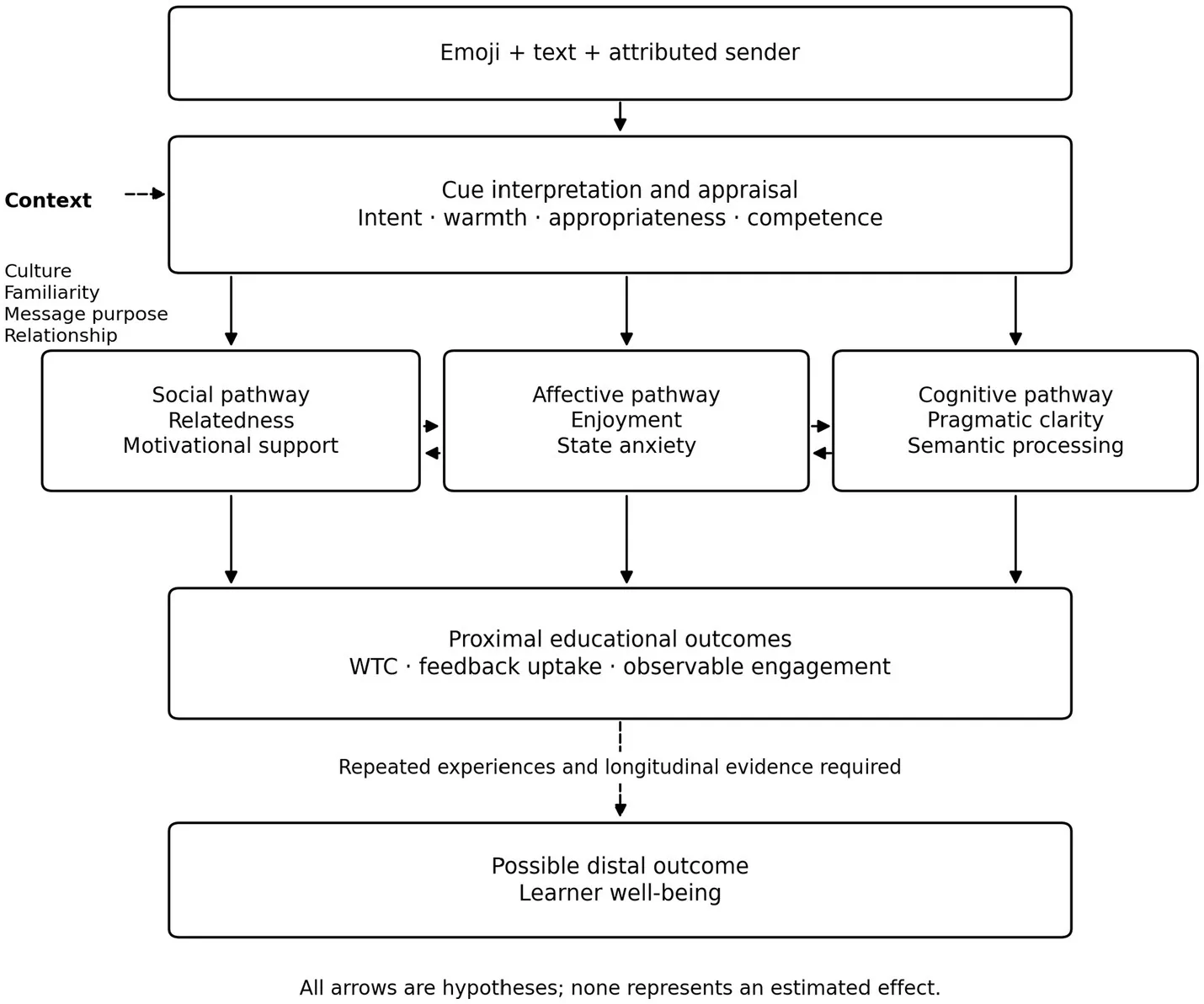
Conditional pathways from emoji-mediated instructional communication to proximal outcomes and possible learner well-being. Solid arrows indicate proposed relations; dashed arrows mark contextual conditioning or a distal extension. Two-way links represent possible interactions between proximal processes. All relationships require empirical testing in the relevant EFL context. WTC, willingness to communicate.

The social pathway links perceived responsiveness and warmth to possible relatedness satisfaction and then to motivation for participation. The affective pathway links the appraisal of an exchange to immediate enjoyment, reassurance, or anxiety. These pathways may interact: feeling understood may make an interaction more enjoyable, while feeling anxious may affect how the next message is interpreted. They should nevertheless be measured separately. Neither perceived warmth nor reported enjoyment is sufficient to infer that a learner feels connected to a teacher.

A cognitive-linguistic pathway concerns interpretation of task meaning, pragmatic force, or visual-semantic correspondence. Improved clarity might reduce uncertainty and thereby influence affect, but an immediate processing advantage could also occur without any change in relatedness. Conversely, an ambiguous cue could increase cognitive effort despite a friendly intention. The model therefore treats cognitive-linguistic processing as a partly independent route that can interact with the socio-emotional routes, rather than assuming that all routes express the same underlying benefit.

These proximal processes may influence WTC, feedback uptake, and observable engagement. Sustained supportive experiences could subsequently be associated with well-being, but this distal link requires repeated measurement, an appropriate comparison, and control for broader instructional and life circumstances. The framework makes no direct, established link from emoji presence to flourishing, resilience, or long-term emotional change. Figure 1 presents hypothesized relations rather than estimated effects.

The model’s contribution is to specify an interpretive entry point, distinguish relatedness from affect and cognitive processing, and locate moderators on those relationships. It also identifies competing outcomes, such as increased caring impressions alongside diminished competence impressions. In this form, a null or adverse effect is theoretically informative: it may indicate that the cue was not noticed, was judged inappropriate, did not change relatedness, or did not translate into participation. Combining theories becomes useful only insofar as it helps distinguish these explanations empirically.

## 7 Pedagogical implications

The framework supports deliberate, context-sensitive communication rather than a recommendation to increase emoji frequency. An instructor considering a cue should first identify its communicative purpose, assess whether learners are likely to share the intended interpretation, and consider the seriousness of the message and the established relationship. Clear wording remains essential. Christophel’s (1990) findings on teacher immediacy provide broader background for attending to relational communication, but they do not establish the effectiveness of emoji use.

For routine encouragement or formative feedback, an emoji may supplement an explicit acknowledgement of effort and a clear next step. It should not substitute for an explanation of an error, a marking criterion, or substantive academic support. Formal decisions, distress disclosures, and sensitive disputes warrant particular care because playful or celebratory cues may conflict with the message. If a cue is misunderstood, clarification of the wording and intent is more useful than insisting that its meaning is obvious.

Cross-cultural variability also suggests attention to emoji literacy: the ability to interpret and deploy visual cues in context. Activities can ask learners to compare the same message with different cues, explain alternative readings, or rewrite the message so that its intention is clear without an emoji. Such tasks are compatible with intercultural communicative competence objectives (Byram, 1997), but gains in that competence should be assessed rather than presumed. Discussion should invite learners’ own interpretations and avoid assigning a single meaning to an entire culture or generation.

Instructors can establish shared communication expectations while allowing students to prefer text-only interaction. Decisions should take account of learner age, proficiency, accessibility, institutional norms, and differences between public group messages and private feedback. Platform developers should preserve the readability of the verbal message and permit control over expressive features. AI-generated feedback should identify its automated source and be reviewed for congruence between wording and visual cues; an emoji must not be treated as evidence of empathetic understanding by the system.

## 8 Specific propositions and future research

### 8.1 Testable relationships

The following propositions concern conditional effects and remain untested as an integrated EFL model. Each requires an explicit comparison and separate measurement of its proposed mechanism and outcome.

P1. Compared with an otherwise identical text-only message, an emoji pretested as appropriate and congruent with the message will increase perceived teacher warmth in a low-stakes instructional exchange. This expectation should be evaluated alongside competence and trustworthiness, which may respond differently. A condition expressing equivalent warmth verbally would test whether the emoji adds anything beyond supportive wording.

P2. Any relationship between emoji-enhanced teacher feedback and subsequent participation will be partly explained by perceived responsiveness and relatedness satisfaction. Relatedness should be measured after exposure and before the later participation outcome. Because mediator–outcome associations are not randomized automatically by randomizing the message, statistical mediation alone would not establish the complete causal mechanism.

P3. A cue interpreted as supportive will be associated with more favourable immediate affect and, potentially, lower state communicative anxiety. A subsequent effect on WTC is expected only where those responses make initiating communication more acceptable to the learner. Enjoyment, state anxiety, WTC, and actual responses should be assessed separately; no prediction is made that they must all change together.

P4. Familiarity, perceived message–emoji congruence, and norms about instructional formality will moderate cue interpretation and its consequences. Effects may be attenuated or reversed when the cue is unfamiliar or judged inappropriate. Cultural background should be examined together with these measured variables rather than used as a sufficient explanation in itself.

P5. Sender attribution will condition responses to an otherwise identical cue. Teacher, peer, and automated-source messages should be compared without assuming that their effects follow a fixed ranking. For object or symbol cues used in a learning task, immediate comprehension and delayed retention should be evaluated independently from affect and relatedness; an immediate semantic benefit need not imply a socio-emotional benefit.

P6. Repeated supportive exchanges may be associated with later well-being through sustained relatedness and engagement, but an isolated emoji exposure is not expected to establish this distal outcome. Longitudinal studies must measure well-being directly, assess baseline differences, and examine alternative explanations such as instructor support, task success, and experiences outside the course.

### 8.2 Designs capable of testing the model

An initial randomized message experiment could compare identical feedback without an emoji, with a locally pretested congruent emoji, and with a potentially incongruent cue. Feedback accuracy, wording, length, grade, sender identity, and response timing should be held constant as far as possible. An additional verbally warm condition would help isolate the visual cue’s incremental contribution. Manipulation checks should assess whether learners noticed the cue and how they interpreted it, including warmth, appropriateness, clarity, and perceived competence. Actual revision quality or a voluntary follow-up response would provide behavioural outcomes beyond favourable ratings.

Subsequent classroom studies could use repeated, low-stakes feedback episodes, with analyses accounting for observations nested within students and classes. Baseline anxiety, proficiency, relationship history, and emoji familiarity should be measured before exposure. Short-term emotion measures can be combined with later participation and validated well-being measures, while qualitative interviews or stimulated recall can examine why the same cue was read differently. Measures used across languages or groups require checks of translation quality and comparability. Sample sizes should be justified through an *a priori* power analysis for the planned contrasts or interactions.

Studies should preregister their principal contrasts and outcomes, report effect estimates with uncertainty, and retain adverse and null results. Longitudinal designs must distinguish changes within learners from stable differences between learners. Until such evidence is available, the model is best used to organize questions and explain boundary conditions, rather than to prescribe a generally beneficial emoji intervention.

## 9 Limitations of the review

This purposive narrative review is vulnerable to selection bias and cannot establish the completeness or relative prevalence of favourable, null, and adverse findings. The targeted update privileges English-language scholarship and sources retrievable through publisher and scholarly web records. Its qualitative appraisal does not replace a systematic risk-of-bias assessment. Heterogeneity in cue type, context, design, and outcome also prevents a defensible pooled estimate from the selected literature.

Much of the evidence concerns immediate judgements, self-reported responses, or populations outside EFL. Older emoticon findings cannot be transferred without qualification to contemporary emojis, and platform conventions change over time. The proposed pathway from interpretation to relatedness, affect, participation, and well-being has not been tested as a whole. The framework should therefore be evaluated against alternative explanations, including supportive wording, prior teacher credibility, novelty, and message comprehension, before stronger pedagogical or well-being claims are made.

## 10 Conclusion

This narrative conceptual review positions emojis as context-dependent socio-emotional cues in digitally mediated EFL instruction. The available literature supports specific claims about message interpretation, impressions, self-efficacy, and immediate task performance in particular settings. It does not establish that emojis improve EFL learner well-being, resilience, or flourishing. Their educational significance depends on how learners interpret the cue in relation to the message, sender, and communicative context.

SDT provides the principal account of possible relatedness and motivational processes, while digital semiotics explains interpretation, broaden-and-build theory motivates a conditional affective extension, and PERMA clarifies the breadth of potential well-being outcomes. By distinguishing these roles and separating proximal from distal outcomes, the model offers a more precise basis for empirical investigation. The pedagogical implication is to attend to learners’ interpretations and the quality of support conveyed, with emojis considered one optional component of clear and responsive communication.
